# Reassessing the Role of *Entamoeba gingivalis* in Periodontitis

**DOI:** 10.3389/fcimb.2018.00379

**Published:** 2018-10-29

**Authors:** Mark Bonner, Manuel Fresno, Núria Gironès, Nancy Guillén, Julien Santi-Rocca

**Affiliations:** ^1^International Institute of Periodontology Victoriaville, QC, Canada; ^2^Centro de Biología Molecular Severo Ochoa, Consejo Superior de Investigaciones Científicas, Universidad Autónoma de Madrid, Madrid, Spain; ^3^Instituto de Investigación Sanitaria del Hospital Universitario de La Princesa, Madrid, Spain; ^4^Institut Pasteur, Paris, France; ^5^Centre National de la Recherche Scientifique, CNRS-ERL9195, Paris, France; ^6^Science and Healthcare for Oral Welfare, Toulouse, France

**Keywords:** *Entamoeba gingivalis*, periodontitis, gingivitis, inflammation, parasitic infection, infectious disease

## Abstract

The protozoan *Entamoeba gingivalis* resides in the oral cavity and is frequently observed in the periodontal pockets of humans and pets. This species of *Entamoeba* is closely related to the human pathogen *Entamoeba histolytica*, the agent of amoebiasis. Although *E. gingivalis* is highly enriched in people with periodontitis (a disease in which inflammation and bone loss correlate with changes in the microbial flora), the potential role of this protozoan in oral infectious diseases is not known. Periodontitis affects half the adult population in the world, eventually leads to edentulism, and has been linked to other pathologies, like diabetes and cardiovascular diseases. As aging is a risk factor for the disorder, it is considered an inevitable physiological process, even though it can be prevented and cured. However, the impact of periodontitis on the patient's health and quality of life, as well as its economic burden, are underestimated. Commonly accepted models explain the progression from health to gingivitis and then periodontitis by a gradual change in the identity and proportion of bacterial microorganisms in the gingival crevices. Though not pathognomonic, inflammation is always present in periodontitis. The recruitment of leukocytes to inflamed gums and their passage to the periodontal pocket lumen are speculated to fuel both tissue destruction and the development of the flora. The individual contribution to the disease of each bacterial species is difficult to establish and the eventual role of protozoa in the fate of this disease has been ignored. Following recent scientific findings, we discuss the relevance of these data and propose that the status of *E. gingivalis* be reconsidered as a potential pathogen contributing to periodontitis.

## Introduction: a disease with underestimated impact

Periodontitis is a disease leading to alveolar bone destruction and eventually tooth loss. The prevalence of periodontitis is constant among the defined World Health Organization (WHO) regions, with around one person out of two between 35 and 44 years old (Petersen and Ogawa, 2005). This prevalence increases with age (Demmer and Papapanou, 2010). In the USA, between 2009 and 2012, 46% of adults aged 30 years or more suffer from periodontitis (Eke et al., 2015).

Periodontitis is a handicapping disease, for which the WHO calculated the loss of 3,518,002 DALYs (Disability-adjusted life years, a measure of disease burden as the loss of healthy life years) in 2015 in the world, meaning 0.132% of the worldwide DALYs (Organization, 2016), though the associated disability weight is low (0.007), reflecting only “minor bleeding of the gums from time to time, with mild discomfort” (Evaluation, 2016). Though periodontitis is linked to systemic diseases like diabetes (Nascimento et al., 2018) and ischemic stroke (Leira et al., 2017), the etiological link is difficult to demonstrate and the possible impact of periodontitis on other ailments is ignored for the calculation of DALYs.

The etiology of periodontitis is still unclear and it is classified by the WHO as a non-communicable disease. Some human genetic factors linked to periodontitis were demonstrated (Vieira and Albandar, 2014) and are still investigated to explain its prevalence in the global population, in conjunction with age (Demmer and Papapanou, 2010). Modifiable risk factors for the disease were also sought and some parameters have been identified: smoking (Eke et al., 2016), alcohol consumption (Wang et al., 2016), and poor oral hygiene (Lertpimonchai et al., 2017). Beyond pain from wounds, eventual edentulism, and defects in occlusion, the patients experience halitosis (Silva et al., 2017) and esthetic issues (Nieri et al., 2013). Altogether, these factors may account for their psychological and social distress (Lopez et al., 2012; Hsu et al., 2015; Dumitrescu, 2016; Reynolds and Duane, 2018).

Evolution toward gum disease goes through three stages (i) formation of dental plaque; (ii) gingivitis, which is an inflammation of the gums due to the dental plaque, and (iii) periodontitis, in which alveolar bone and fibers that hold the teeth in place are irreversibly damaged. The pathophysiology of the disease is harshly debated, but a consensus was reached about some key points. First, inflammation is compulsory and prior to bone loss, evidenced by pocket formation [reviewed in Van Dyke (2017)]. Second, the microbial flora in periodontal pockets differs from that observed in healthy sulci (Marsh, 1994). Last, plaque and calculi worsen prognosis (Löe et al., 1965). Consensual treatment in clinics is thus based on the mechanical and/or surgical removal of dental plaque, calculi, and damaged/inflamed tissues (Smiley et al., 2015). These paths lead to an inefficient solution dealing with late symptoms without considering the evoked causes of the disease. The keystone for the improvement of periodontitis management worldwide is a better knowledge of its pathophysiology.

Due to the important correlation of periodontitis with the presence of *Entamoeba gingivalis* in the oral cavity, here, we searched for the facts that can shed light on the question of whether *E. gingivalis* plays a role in the occurrence of the periodontitis. In this review, we summarize existing data on the biology of the amoeba *Entamoeba gingivalis* and on its potential role as an infectious agent in periodontitis. We aim at highlighting perspectives for new research on the pathophysiology and prophylaxis of this neglected disease.

## Microbiology of periodontitis: the bacterial paradigm

Though the saliva contains low nutrient concentrations and antimicrobial defense systems [reviewed in van 'T Hof et al. (2014)], the healthy oral cavity houses a commensal microbiota, composed of bacterial communities [about 1,000 species across humans, Consortium (2012)], whereas the contribution of viruses, parasites, archaea, and fungi is still to be characterized. Microorganisms and oral mucosae maintain a mutualistic, resilient symbiotic relationship (Rosier et al., 2018). The bacterial ecosystem of healthy sulci is intriguingly similar between individuals and it comprises immotile bacilli and cocci, as seen in microscopy (Listgarten, 1976), with bacterial species differing from those encountered on the tongue (Aas et al., 2005; Consortium, 2012; Rogers and Bruce, 2012). At the tooth surface, in particular in the dental sulcus, nutrients coming from food and cellular debris accumulate and support the survival of bacteria that adhere and colonize the dental enamel. Bacterial flagella, pili, and wall proteins can recruit other bacteria, by co-aggregation (Kolenbrander and Celesk, 1983; Gibbons et al., 1988). Furthermore, the secretion of polysaccharides initiates the formation and organization of a scaffold (Jakubovics, 2010), while intercellular signaling molecules regulate biofilm development, in particular through a *quorum sensing* mechanism mediated by different types of messengers, as cyclic di-guanosine monophosphate or LuxS [reviewed in Marsh et al. (2011)]. This intra- and inter-species communication leads to coordination of activities and increases the chances of genetic material transfer. The resulting dental plaque is an organized biofilm, whose formation is not pathologic (Gibbons and Van Houte, 1973), though it was thought to be responsible for gingivitis and periodontitis (Schultz-Haudt et al., 1954).

Some bacteria are associated with periodontitis and this led to the proposal of a specific plaque explanation for the disease (Loesche, 1979). These bacteria group in clusters associated with disease progression (Socransky et al., 1998), reflecting the sequential colonization of the periodontal sulcus and pocket (Li et al., 2004; Feres et al., 2016). The “periodontopathogenic” red complex is comprised of anaerobic bacteria (*Aggregatibacter actinomycetemcomitans, Tannerella forsythia*, and *Porphyromonas gingivalis*), supporting the hypothesis of a gradual modification of the environment prior to and necessary for colonization by other bacterium types (Marsh, 2003; Darveau, 2010). However, *P. gingivalis* is present in some healthy patients (Socransky et al., 1998) and is not abundant, even in periodontitis (Moore et al., 1982), while this “keystone pathogen” provokes environmental changes in the sulcus promoting inflammation (Hajishengallis et al., 2011). Thus, *P. gingivalis* cannot be considered an etiological agent for periodontitis by itself, at least with respect to Koch's postulates.

Koch's postulates are the extreme case of Hill's criteria for causation (Hill, 1965) in which infection by a single etiologic agent is the unique parameter influencing the occurrence of the disease (Inglis, 2007). Thus, the quest for a single pathogen explaining the etiology of periodontitis by itself, following Koch's postulates, may be in vain. Contrariwise, periodontitis, as a biofilm disease (Schaudinn et al., 2009), may result from the integration of various causative parameters. Bacteria are among these parameters and the composition of the microbial communities accurately correlates with clinical outcome (Feres et al., 2016; Hunter et al., 2016). Indeed, some species can be efficiently used as markers for diagnosis (Meuric et al., 2017), re-opening ways for considerations about the use of bacterial identification–though in a multi-variate fashion–for epidemiology or treatment follow-up.

Beside changes in its composition, the bacterial community can harbor changes in its functions, generating a new equilibrium (dysbiosis) that is possible in the new dental plaque environment (Hajishengallis and Lamont, 2012). Bacterial entities collaborate and functional changes, such as synergism, are evidenced at the transcriptional level (Kirst et al., 2015; Yost et al., 2015; Deng et al., 2018). Nevertheless, the abundance of some bacterium species is not synonymous for their activity (Mark Welch et al., 2016), a fact compatible with the “keystone pathogen” theory and the role of *P. gingivalis* in shaping the ecology of the periodontal pocket. This ecology is impacted by the dysbiotic communities, as evidenced *in vitro* with deregulated host inflammatory responses (Yost et al., 2017; Herrero et al., 2018). The consequences of dysbiosis were also evidenced *in vivo* at the systemic level, with metabolic changes linked to diabetes (Branchereau et al., 2016; Blasco-Baque et al., 2017).

## Immuno-pathological processes TOWARD periodontitis

While the dental plaque stacks, mineralization leads to formation of tartar deposits, which can cause injury, as well as overhanging restorations or repetitive wounding. In parallel, the constant presence of bacterial components and the possible colonization by periodontopathogens can be sensed by the host and can cause chronic inflammation and an initial tissue lesion [reviewed in Kurgan and Kantarci (2018)]. The host takes part in fueling progression to disease by inflammation and active mediators of inflammation resolution improve the disease's outcome (Lee et al., 2016; Mizraji et al., 2018). Resident leukocytes and endothelial cells respond to bacterial biofilms: vascular permeability increases and interleukin 8 attracts neutrophils to the affected tissues (Tonetti et al., 1998). Neutrophils play a pivotal role in periodontitis (Ryder, 2010), producing reactive oxygen species (ROS), with probable impact at the systemic level [reviewed in Wang et al. (2017)]. While the early lesion progresses, other immune cells are recruited, including macrophages, lymphocytes, plasma cells, and mast cells. The inflammation can be visible at the microscopic level, where rete pegs–epithelial projections into the underlying connective tissue–form in the pocket epithelium and blood vessels proliferate [reviewed in Zoellner et al. (2002)], and at the macroscopic level, in particular by reddening and bleeding. Macrophages are predominant at this stage (Dennison and Van Dyke, 1997); they can derive from circulating monocytes produced in the spleen or be resident macrophages from embryonic origin, like Langerhans cells, with a possible different role during gum inflammation (Moughal et al., 1992). In established lesions, adaptive responses take place and lymphocytes are abundantly detected (Gemmell et al., 2007). Collagen fibers are increasingly altered, leading to a severe tissue remodeling and loosening of the pocket epithelium (Payne et al., 1975). Thus, greater amounts of dental plaque can accumulate in subgingival locations and aggravate gingivitis, which is considered reversible after elimination of the biofilm, in particular due to the absence of bone and periodontal ligament destruction (Ebersole et al., 2017).

Left untreated, gingivitis evolves to periodontitis, which is characterized by an inflammatory infiltrate composed of plasma cells, and by degradation of collagen fibers, loss of connective tissue, and bone destruction in an anaerobic environment (Mettraux et al., 1984). Periodontitis results in clinical attachment loss, i.e., the deeper positioning of the junction between the pocket epithelium and the cementum of the tooth root. The resulting volume below the gum forms the periodontal pocket, witnessing the breaking down of the initial epithelial attachment, the destruction of the connective tissues constituting the periodontal ligament, and the lysis of the alveolar bone. The sulcus depth–the distance between the free gingival margin and the epithelial attachment–is inferior to 3 mm in healthy or gingivitis sites. When superior to 3 mm, periodontitis is suspected and confirmed by inflammation (redness and swelling) and bleeding on probing.

During periodontitis, the balance between bone resorption and regeneration is displaced: Th17 cells induce osteoclastogenesis (Sato et al., 2006). Recently, it has been described that immunopathogenic Th17 lymphocytes [converted from Foxp3+ T cells; a recent review on T cell contribution to periodontitis in Kinane et al. (2017)] that cause bone damage in rheumatoid arthritis can also determine bone resorption and antimicrobial immunity in the oral cavity (Tsukasaki et al., 2018). In human periodontal lesions, Th17 lymphocytes are abundant (Hajishengallis, 2014) and the major source of IL-17. Foxp3+IL-17+ cells are found in the transition state. The generation of pathogenic exFoxp3+TH17 cells in the oral mucosa is dependent on IL-6, which is expressed by periodontal ligament fibroblasts in periodontitis and stimulated by bacterial PAMPs as LPS (Yamaji et al., 1995) through PRRs as TLR-2 and -4 (Sun et al., 2010; Makkawi et al., 2017). Furthermore, osteogenesis is impaired during periodontitis, while bone resorption by osteoclasts is promoted (Zhou et al., 2018), highlighting that not only immune mechanisms are involved in periodontitis pathophysiology. Beside the bacteria and human cells, some archaea, viruses, protozoa, and fungi are differentially present in healthy and diseased sites (Deng et al., 2017). The contributions of these different phenomena, as well as lysis by pathogens or other host immune cells, still need to be elucidated to solve the current paradigm of periodontitis physiopathology, in which only some of the players are visible in the game.

## *Entamoeba gingivalis* and periodontal infections

### Debate about the presence of *Entamoeba gingivalis* during periodontitis

#### Discovery of *Entamoeba gingivalis*

Though periodontitis was described since antiquity (Langsjoen, 1998), its association with parasites has been evidenced only a century ago. The first description of *Entamoeba gingivalis*–then named “*Amoebea gengivalis*”–was laconically performed in 1849 from dental plaque samples, mentioning amoebic movement and the presence of internal vesicles (Gros, 1849). Though free amoebae were described since 1755 (Rösel Von Rosenhof, 1755), *E. gingivalis* is the first amoeba which was found in humans. The pathogenic association of amoebae with humans was first documented in 1875, validating Koch's postulates in an animal model for the pathogen *Entamoeba histolytica*, then named “*Ameba coli*” (Lösch, 1875). The pathogenicity of *Entamoeba gingivalis* was questioned early (Kartulis, 1893) and the first systematic study associating it with periodontitis was preliminarily published in 1914 (Barrett, 1914): amoebae were detected in the totality of the 46 cases of pyorrhea (periodontitis) that were enrolled in the study. The authors later included 7 healthy individuals from the same group of patients in the “Insane Department of the Philadelphia Hospital” and claimed they could not detect amoebae in “the detritus collected around the neck of the teeth” (Smith and Barrett, 1915a). Furthermore, administration of emetine caused the withdrawal of amoebae and was followed by the cure of pyorrhea in 13 patients (Barrett, 1914). As emetine was thought to be a specific amoebicidal alkaloid with poor bactericidal effect, the etiological link between *Entamoeba gingivalis* and periodontitis was extrapolated and led Smith and Barrett to rename the disease “amoebic pyorrhea” (Barrett, 1914) or “oral endamebiasis” (Smith and Barrett, 1915b).

#### Rejection of amoebic etiology for periodontitis

It is of epistemological importance to underline that systematic studies, some with a low number of participants, about the involvement of *Entamoeba gingivalis* in periodontitis were countered by specialist opinions without formal experimental proofs, as reported by Craig (Craig, 1916). This controversy led to an almost total abandon of the etiology- and emetine-based therapy, discrediting at the same time its original scientific background. Further statements about the non-permanent cure were made but were unsupported by scientific data, and incriminating relapses (Howitt, 1925). However, the possibility of re-infections was completely ignored and casts doubts about the understanding of both pathophysiology and epidemiology of the disease at that time. In parallel, a method for the culture of *E. gingivalis* was described, which allowed the study of the effects of emetine hydrochloride on the parasite: “emetine hydrochloride has, apparently, no very marked amoebicidal action *in vitro* against either of the strains of *E. gingivalis* used” (Howitt, 1925). However, in a single experiment were evaluated the minimum lethal concentration (MLC, around 116.5 μM, 1:16,600 dilution) and the subjectively determined minimal inhibitory concentration (MIC, around 38.5 μM, 1:50,000) were evaluated. It is noteworthy that the MLC obtained for the emetine-sensitive HM1:IMSS *E. histolytica* strain in a more recent study is higher than 100 μM, the IC_50_ is 29.9 μM, and the MIC is lower than 1 μM (Bansal et al., 2004). This underlines that the experimental methodology to study the effect of emetine on *E. gingivalis* was not accurate. The conclusions of this paper should thus be considered with caution because this *in vitro* study did not corroborate the results reported during the treatment of patients, as previously cited.

#### *Entamoeba gingivalis* infections in the genetic Era

After this controversy, only a few studies based on the microscopic detection of *E. gingivalis* were published, but almost all of them revealed a prevalence of the parasite close to 100% in advanced periodontal pockets (Fisher, 1927; Hinshaw and Simonton, 1928; Wantland and Wantland, 1960; Wantland and Lauer, 1970; Gottlieb and Miller, 1971; Keyes and Rams, 1983; Lange, 1984; Linke et al., 1989). In the cited publications, the prevalence of *E. gingivalis* in healthy sulci–when studied–ranged from 0 to 26%, suggesting possible errors in the identification of the amoeba. Development of gene amplification by polymerase chain reaction (PCR) and the sequencing of a gene of *E. gingivalis* (Yamamoto et al., 1995) opened ways for the molecular identification of the parasite and accurate epidemiological studies. The first study–using a long amplicon (1.4 kb) and a sub-optimal DNA purification protocol–revealed a prevalence of 6.25% (2 sites out of 32, from 8 patients) in gingivitis or periodontitis sites, without precision of either their relative number or grade; no amplification was obtained from 20 healthy sites (Kikuta et al., 1996). In the second study, 69.2% of periodontitis sites were positive in real-time PCR for *E. gingivalis*, while none of the 12 healthy sites included in the study were (Trim et al., 2011). A third study showed a prevalence of 80.6% (58/72) in periodontitis sites and 33.3% (11/33) in healthy sites by conventional PCR, with controls of PCR inhibition and matrix degradation (Bonner et al., 2014). Recently, transcriptomics revealed that *E. gingivalis* 18s rRNA sequence was detected in all (4/4) periodontal pockets and was less abundant in 60% (6/10), or undetected in 40% (4/10) of healthy sites (Deng et al., 2017). Furthermore, genetic variants of *E. gingivalis* have been identified (Cembranelli et al., 2013; Garcia et al., 2018b) and different levels of virulence reflected at the transcriptomic levels in genetically identical parasites (Santi-Rocca et al., 2008) may account for discrepancies in their molecular detection as compared with microscopy or clinical diagnoses. The new clinical characterization of periodontitis (Tonetti et al., 2018) will avoid further confusion about the definition of health and various disease grades, that may also be responsible for variability between the studies.

Altogether, these data suggest that *E. gingivalis* may be asymptomatically present in some sulci and may be associated with the disease after environmental changes, reminiscent of the intestinal pathogen *E. histolytica*.

### Life cycle of *E. gingivalis*

In most species of the genus *Entamoeba*, two cellular forms have been identified in nature: the cyst, which is the contaminant form found in the environment, and trophozoites, the vegetative cell able to divide, that initially derives from excystation of cysts ingested by the host. The survival of these *Entamoeba* species is ensured by their encystment in response to environmental changes (Mi-Ichi et al., 2016), permitting the survival in environments exposed to oxygen, like human stools, where identification of *Entamoeba* species is made by a simple morphological phenotyping that relies on the number of nuclei carried by the cyst. The sole *E. gingivalis* would not encyst, though cysts of *E. gingivalis* were reported in the literature at the beginning of the twentieth century (Chiavaro, 1914; Smith and Barrett, 1915b; Craig, 1916). However, it is now commonly accepted that *E. gingivalis* does not produce cysts, considering the absence of proof as a proof of absence. Nevertheless, the parasite *E. gingivalis* is essentially observed in periodontal pockets, suggesting that low oxygen levels are important for the survival of trophozoites, as in the case of *E. histolytica* and *E. dispar* [reviewed in Olivos-Garcia et al. (2016)]. Direct transmission of trophozoites to a new host would imply that they are resistant to oxygen, which raises questions about how *E. gingivalis* is transmitted in nature, and what ecological niche serves as a reservoir for this microorganism. Unfortunately, the complete life cycle of *E. gingivalis* is still missing and not addressed yet, hampering efficient prophylaxis.

In the closely-related specie *E. histolytica*, resistance to oxygen is modulated by interaction with bacteria (Varet et al., 2018), as well as virulence (Bracha and Mirelman, 1984; Galvan-Moroyoqui et al., 2008). The microbiota could be of major importance in switching from commensal to pathogenic forms and explain why only a minor part of *E. histolytica* intestinal infections are invasive and symptomatic. During periodontitis, bacterial virulence genes are strongly modulated (Deng et al., 2017) and the frequent and abundant detection of *E. gingivalis* in periodontitis pockets (Bonner et al., 2014) suggests and warns that interactions between constituents of the microbiota could be essential for their functions during the pathophysiology of the disease.

### Ingestion of human cells by *E. gingivalis*

*Entamoeba gingivalis* resembles *E. histolytica* in several aspects: trophozoites measure about 30 μm, they are both endowed with mobility, and they ingest human cells and bacteria. The ability of *E. histolytica* to kill and phagocytose host cells correlates with parasite virulence and this amoeba is able to feed on human cells: erythrocytes, lymphocytes, and epithelial cells (Christy and Petri, 2011). Two mechanisms of cell killing and uptake have been discovered for *E. histolytica*: phagocytosis and trogocytosis (Ralston et al., 2014).

Phagocytosis is the phenomenon by which single cells ingest large volumes of material, like other cells or big particles; phagocytic cells include diverse unicellular entities as amoebae, but also macrophages and neutrophils that are cells from the immune system [a recent review in Niedergang and Grinstein (2018)]. In *E. histolytica*, phagocytosis is indispensable for its nutritional needs since this amoeba ingests bacteria in the intestinal lumen. Moreover, phagocytosis is correlated with virulence because *E. histolytica* kills human cells that are eventually phagocytosed. The current model is that *E. histolytica* first kills the host cells in a contact-dependent manner and then phagocytosis of dead cells takes place. However, live cells like bacteria and erythrocytes are also phagocytosed by *E. histolytica*.

In a recently-discovered second mechanism for cell damage, *E. histolytica* ingests fragments of live host cells in a nibbling-like process termed “trogocytosis” (Ralston et al., 2014). Though a specific AGC kinase1 was found exclusively involved in trogocytosis (Somlata and Nozaki, 2017), it seems premature to completely dissociate them from the phagocytic process. Furthermore, previous evidence has shown the existence of these structures during phagocytosis of epithelial and endothelial cells by *E. histolytica* [(Lejeune and Gicquaud, 1987; Nakada-Tsukui et al., 2009), Figure 2 in Faust et al. (2011)] and variability of phagocytic cup shape exist during phagocytosis (Tollis et al., 2010). It has been suggested that amoebic trogocytosis essentially concerns bits of live cells that are internalized, and phagocytosis is the process by which an entire cell is internalized (Ralston, 2015).

**Figure 1:**
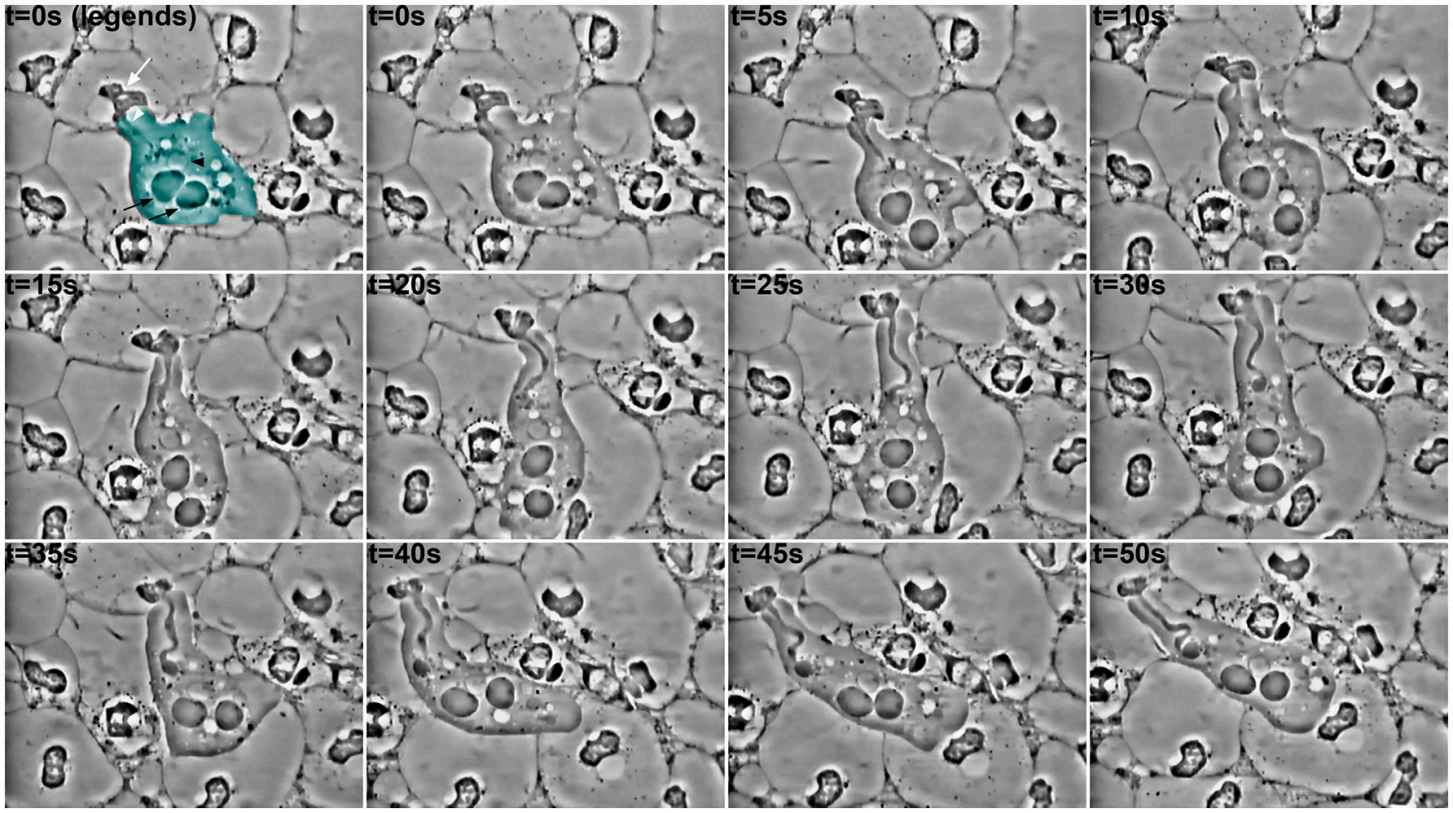
Ingestion of material by an amoeba in a periodontal pocket. Pictures extracted every 5 s from a video-microscopy of saliva-mounted plaque from the deepest part of the periodontal pocket, at 1,000 × magnification. In the first panel, the amoeba is pseudo colored in cyan; a black arrowhead indicates its nucleus with the typical peripheral chromatin, while the central karyosome is out of focus. The black arrows indicate food vacuoles. The white arrow designates the internal material (possibly a modified nucleus, perhaps with other subcellular structures) from a host cell (probably a leukocyte), whose ingestion has begun through a “channel,” as already observed for trogocytosis and erythrophagocytosis in *E. histolytica*. After 30 s, a food vacuole begins to form at the extremity of the channel. It is noteworthy that the amoeba continues to emit pseudopods and to move during the process, and that it is surrounded by cells with nuclei of different shapes, or even lacking.

Phagocytosis of parts of human cells by *E. gingivalis* was reported almost a century ago (Child, 1926) but poorly studied since then. Some exceptions however existed and, thanks to video microscopy, the impressive process of cell ingestion by this amoeba has been highlighted (Lyons and Stanfield, 1989; Bonner et al., 2014). *Entamoeba gingivalis* is able to engulf one or more human cells at the time by a yet-undescribed mechanism (Figure 1). In the observed samples, cells around the amoebae present an altered cellular content, suggesting *E. gingivalis* can trigger signals leading to modification of human cells. As the only human cells observed in these samples were polymorphonuclear cells, and the literature mentions neutrophils are predominant in periodontal pockets, the target cells of amoebic phagocytosis may be the latter. The processes leading to the modifications in nuclear and cytoplasmic morphology in these cells remain to be defined and could be linked, for instance, to proteolytic activity of the amoebae and bacteria, or to a delayed/frustrated NETosis (Neutrophil extracellular traps). Whatever this process is, and whether the amoeba phagocytoses or trogocytosis, the cellular content of the neutrophils leads to one certain point: the first line of defense of cellular innate immunity against *E. gingivalis* and other organisms in the dysbiotic biofilm lacks its weapon (nuclei for NET formation and gene expression) and is thus unable to accomplish its functions.

### Hot topics about *E. gingivalis* infections

The parasite *E. gingivalis* was identified for its amoeboid movement (Gros, 1849) and characteristics of its locomotion have not been studied since, unlike the closely-related species *E. histolytica* (Aguilar-Rojas et al., 2016). While observations in wet-mounted slides are possible, culture of the parasite is still a bottleneck for the study of *E. gingivalis* biology. Division events have not recently been documented and the whole life cycle is yet to be described.

The genetic variability in the species *E. gingivalis* seems important (Garcia et al., 2018b) and the genetic distance between the ST1 and ST2 variants may indicate great differences in their biology, accounting for their probable association with different pathologies (Garcia et al., 2018a). As the genotyping of *E. gingivalis* is based on the only gene that has been sequenced, a greater genetic variability may be expected, and further epidemiological studies will precise if some subtypes colonize specific sulcus/pocket environments and, thus, correlate with different clinical outcomes.

The role of the microbiota cannot be ignored and the strong modulation of bacterial genes during periodontitis supports a dysbiotic environment that may impact and be impacted by *E. gingivalis* parasites. Transcriptomic studies will provide clues about these interactions and will rely on the identification of amoebic genetic material. This will be rendered possible by sequencing the *E. gingivalis* genome, probably after the axenic culture of the parasite.

The parasite *E. gingivalis* is more prevalent and more abundant in periodontal pockets, suggesting that this ecological niche is either propitious for its survival, or that the parasite induces changes leading to this environment. Further studies will have to take into consideration the physicochemical and biological characteristics of the periodontal pockets to allow relevant studies of the biology of the parasites, either *in vitro* or in animal models.

## Conclusion

The absence of reliable animal models for infection by *E. gingivalis* after its axenic culture impedes the ability to conclude about its etiological role in periodontitis, with respect to Koch's postulates. However, recent advances in the field of periodontitis have introduced moderation about this vision, considering that keystone pathogens can participate in changing the environment and consequently in causing dysbiosis, without being the exclusive etiological agent of the disease. *Entamoeba gingivalis* can be an important agent in the pathophysiology of periodontitis and, since its presence is documented and undoubted, it cannot be ignored.

Interestingly, the natural diversity in the human host has allowed the identification of various components of periodontal infections: some pathological traits are preferentially associated with human genetic variants (Offenbacher et al., 2016) and the *P. gingivalis* paradigm may be only one of the possibilities or one of the steps of periodontitis pathophysiology. Indeed, evidence about the kinetics of periodontitis setup is scarce and other possible agents must be considered. First, the ST2 “kamaktli” variant, with a high genetic divergence from *E. gingivalis* ST1 (Garcia et al., 2018b) reminds us that strains of parasites may not be equally virulent or may not have the same tropism (Garcia et al., 2018a). Second, some transcripts from archaea species and viruses are differently abundant between the healthy and the diseased (Deng et al., 2017). In particular, herpes viruses are associated with periodontitis (Slots, 2015; Zhu et al., 2015; Li et al., 2017). Finally, another protozoan is present in some cases of human periodontitis: *Trichomonas tenax* (Marty et al., 2017). Further studies are needed to decipher the ecosystem of the different stages of the periodontal pockets, assigning roles for all of the detected biological entities, which can be opportunistic, neutral, pathogenic, or mutualistic with the organisms within the pocket, and which can have the same type of interactions with the host tissues. Interestingly, the host directly participates in the pathogenic process: osteogenesis is impaired during periodontitis, while bone resorption by osteoclasts is promoted (Zhou et al., 2018). Furthermore, inflammation has an important role in the disease, since active mediators of inflammation resolution improve the disease's outcome (Lee et al., 2016; Mizraji et al., 2018). The contributions of these different phenomena, as well as lysis by pathogens and host immune cells, still need to be elucidated.

All species of *Entamoeba* are not necessarily pathogenic and some of them are commensals (e.g., *E. dispar* or *E. coli*). The study of *E. gingivalis* biology during infection, in particular its virulence factors and pathogenic processes, will allow us to better understand the whole interactions in the ecosystem of the periodontal pockets and to determine the potential participation of *E. gingivalis* in the pathophysiogenesis of periodontitis. Further research should determine if it is taking part in the pathophysiology of periodontitis or just a *bona fide* marker of the disease. In addition, as the picture is getting more complex and the genetic susceptibility of patients shapes different microbiota (Offenbacher et al., 2016), what we call periodontitis might be a group of related diseases with comparable outcomes. Fundamental research must consider this variability to elucidate the pathophysiology of periodontal diseases and to implement efficient public health measures.

## Author contributions

All authors contributed equally to the redaction of the manuscript, conceived, and directed by JS-R.

### Conflict of interest statement

The authors declare that the research was conducted in the absence of any commercial or financial relationships that could be construed as a potential conflict of interest.
